# Impact of Coronavirus Disease on the Ophthalmology Residency Training in Brazil

**DOI:** 10.5935/0004-2749.20210085

**Published:** 2021

**Authors:** Manoela Pessoa de Melo Corrêa Gondim, Pedro Henrique Carneiro, Rafael Moreno, Maria Isabel Lynch

**Affiliations:** 1 Serviço Oftalmológico de Pernambuco, Recife, PE, Brasil.; 2 Hospital das Clínicas, Universidade Federal de Pernambuco, Recife, PE, Brasil.; 3 Universidade de Pernambuco, Recife, PE, Brasil.

Dear Editor,

On February 26, 2020, the Ministry of Health confirmed the first case of the new coronavirus disease (COVID-19) in Brazil^(1)^. Owing to the impact of the disease, higher education institutions were forced to quickly adapt to the new reality and modify their pedagogical plan^(2-5)^. The United Nations International Children’s Emergency Fund reported that the education of an estimated 1.57 billion students in more than 190 countries was interrupted, corresponding to 91% of students worldwide.

This study designed, validated, and implemented an electronic questionnaire for the 95 coordinators of residency courses in ophthalmology that were accredited by the Brazilian Council of Ophthalmology. Through the Google Docs web application, 6 closed-ended questions with predefined responses and 1 open-ended question were prepared, and the link was sent by e-mail and instant messaging application in May and June 2020. For automatic analysis of the responses, the Google Forms platform was used.

**Figure 1:**
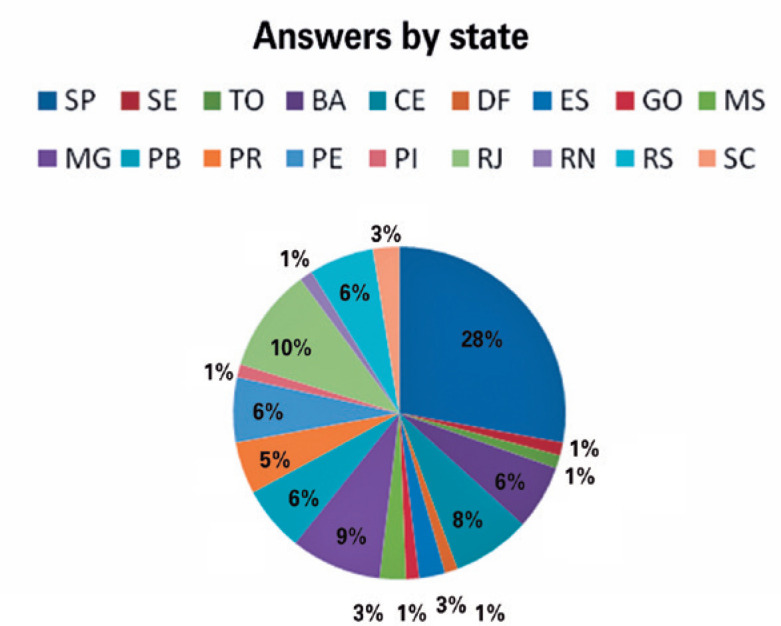
The answers by state.

After sending the questionnaires, 79 responses were obtained in compliance with the Free and Clarified Consent Form. The survey response rates of the course coordinators according to the distribution of the Brazilian regions were as follows: 1.3%, Northern region; 30.4%, Northeast region; 5.1%, Midwest region and Federal District; 49.3%, Southeast region; and 13.9% in Southern region. The answers by state are shown in figure 1.

The coordinators answered that during the pandemic, theoretical activities took place as distance learning in 95% of the courses, attendance increased in 50 courses (63.3%), and the theoretical workload was the same as in face-to-face learning in 19 courses (24.1%) but decreased in 6 (7.6%).

The data collected through the questionnaire regarding surgeries and consultations are shown in table 1.

**Table 1:** All answers regarding consultations and surgeries

	Elective consultations	Elective surgeries	Emergency consultations	Emergency surgeries
Continue normally	1 (1,3%)	1 (1,3%)	18 (22,8%)	17 (21,5%)
Reduced patient numbers	20 (25,3%)	6 (7,6%)	25 (31,6%)	25 (31,6%)
Drastically reduced patient numbers	15 (19%)	16 (20,3%)	28 (35,4%)	30 (38%)
Occasional outpatient care	18 (22,8%)	13 (16,5%)	----	2 (2,5%)
Suspended	25 (31,6%)	43 (54,4%)	8 (10,1%)	5 (6,3%)

Regarding the attendance of students in face-to-face activities in residency training, residents remained with a full workload in 13 services (16.5%) and had a reduced workload in 36 courses (45.6%); however, they were not relocated to help tackle the pandemic. Nonetheless, 21 coordinators (26.6%) responded that in addition to the reduced workload, residents were also assigned in the frontline in the fight against COVID-19.

Coordinators were also asked an open-ended question regarding the existence of any project to replace the disrupted ophthalmological activities during the pandemic. Of the 69 coordinators who responded, 23 (33.3%) still had no plan of action, while 46 (66.6%) reported they had a strategy to recover the time that was lost. Of the 46 coordinators, 8 (17.39%) awaited guidance from higher entities, 5 (10.8%) replied that they planned to extend the period for course completion, 2 (4.3%) intended to promote clinical and surgical task forces as a mitigation measure, and 12 (26.1%) answered that they intended to increase the residents’ workload. No response was obtained for 10 courses (12.7%).

The present study shows that owing to the COVID-19 pandemic, an important change occurred in the dynamics of the ophthalmology teaching courses in Brazil, a similar fact to what is happening in various residency programs worldwide^(2-5)^. This can be clearly seen in the fact that 45.6% of the ophthalmological education services had their students’ workloads reduced to varying degrees, in addition to cases in which the students had to be transferred to the COVID-19 support area. This change in teaching programs may have consequences on the quality of medical training, which must be carefully analyzed.

The impossibility of face-to-face theoretical meetings that is associated with the reduction of practical hours made online theoretical activities indispensable to the maintenance of residency training in ophthalmology. The data collected indicated that 63.3% of the courses managed not only to follow online theoretical activities but also to increase attendance as compared with those during the face-to-face learning pre-pandemic period.

Despite the aforementioned approaches, it is indisputable that in all the courses, the training of residency students had undergone some degree of disruption that will not be easy to make up for. In view of the current world scenario, time should be taken to reflect and reevaluate the pre-pandemic teaching methods in the ophthalmology residency training in Brazil. The existing technology can be much better exploited and improved by faculty members willing to make changes.
